# Prevalence and correlates of home delivery amongst HIV-infected women attending care at a rural public health facility in Coastal Kenya

**DOI:** 10.1371/journal.pone.0194028

**Published:** 2018-03-20

**Authors:** Stevenson K. Chea, Tabitha W. Mwangi, Kennedy K. Ndirangu, Osman A. Abdullahi, Patrick K. Munywoki, Amina Abubakar, Amin S. Hassan

**Affiliations:** 1 Department of Nursing Sciences, School of Health and Human Sciences, Pwani University, Kilifi, Kenya; 2 Department of Public Health, School of Health and Human Sciences, Pwani University, Kilifi, Kenya; 3 Kilifi County Hospital, Department of Health, Kilifi, Kilifi County, Kenya; 4 Centre for Geographic Medicine Research (Coast), Kenya Medical Research Institute, Kilifi, Kenya; 5 Department of Psychiatry, University of Oxford, Oxford, United Kingdom; University of North Carolina at Chapel Hill, UNITED STATES

## Abstract

**Background:**

Home delivery, referring to pregnant women giving birth in the absence of a skilled birth attendant, is a significant contributor to maternal mortality, and is encouragingly reported to be on a decline in the general population in resource limited settings. However, much less is known about home delivery amongst HIV-infected women in sub-Saharan Africa (sSA). We described the prevalence and correlates of home delivery among HIV-infected women attending care at a rural public health facility in Kilifi, Coastal Kenya.

**Methods:**

A cross-sectional design using mixed methods was used. Quantitative data were collected using interviewer-administered questionnaires from HIV-infected women with a recent pregnancy (within 5 years, n = 425), whilst qualitative data were collected using focused group discussions (FGD, n = 5). Data were analysed using logistic regression and a thematic framework approach respectively.

**Results:**

Overall, 108 (25.4%, [95% CI: 21.3–29.8]) participants delivered at home. Correlates of home delivery included lack of formal education (aOR 12.4 [95% CI: 3.4–46.0], p<0.001), history of a previous home delivery (2.7 [95% CI:1.2–6.0], p = 0.019) and being on highly active antiretroviral therapy (HAART, 0.4 [95% CI:0.2–0.8], p = 0.006).Despite a strong endorsement against home delivery, major thematic challenges included consumer-associated barriers, health care provider associated barriers and structural barriers.

**Conclusion:**

A quarter of HIV-infected women delivered at home, which is comparable to estimates reported from the general population in this rural setting, and much lower than estimates from other sSA settings. A tailored package of care targeting women with no formal education and with a history of a previous home delivery, coupled with interventions towards scaling up HAART and improving the quality of maternal care in HIV-infected women may positively contribute to a decline in home delivery and subsequent maternal mortality in this setting.

## Background

Every year an estimated 300,000 maternal deaths occur globally, with 99% of these in developing countries[1]. In 2015, the sub-Saharan Africa (sSA) region alone accounted for approximately 66% of maternal deaths[1], with home deliveries being one of the key contributing factors[2,3]. Unlike in high-income countries, home deliveries in low-income countries occur in the absence of skilled attendants and are therefore associated with increased maternal complications and death[4–7].

Previous studies across the sSA region report variable prevalence of home deliveries ranging from 9% to 94% amongst women in the general population[8–20], and10% to 53% amongst HIV-infected women[21–26].

Risk factors contributing to the poor uptake of hospital delivery among women in the general population have included low socioeconomic status, long distance to nearest health facility, older maternal age, higher birth order, being a Muslim and/or without a religion, low education level, non-attendance of antenatal care, residing in rural areas, poor attitudes of health care providers, lack of transport, inadequate resources in health facilities, having more than two children, being in a polygamous marriage, and perceived poor quality of care during delivery in health facilities[8–12,17–20,27–30]. Social and cultural factors including previous successful home deliveries, belief that local herbs speed up labour, belief that women should exhibit endurance during child birth, lack of participation in decision making, ready availability of traditional birth attendants(TBA), and presence of male midwifes in health facilities have also been identified as deterrents to hospital deliveries[8,12,28,30].

Risk factors contributing to home deliveries among HIV infected women are comparable to those in women in general population and included low socioeconomic status, long distance to nearest health facility, HIV related stigma, security concerns when a woman goes into labor at night, low education level, fear of harsh treatment at the health facility, not attending antenatal clinic(ANC), not using antiretroviral drugs(ARVs), not knowing partner’s HIV status, unsatisfactory delivery care at the health facility and high parity[4,5,22,25,26,31]. Cultural factors identified include lack of commitment from husband, a feeling that facility delivery is not necessary if previous births were free of problems, previous successful home birth, encouragement by grandmothers to have a home birth, rapid progression of labor and preference to be assisted by a TBA[4,5,26,31].

A recent study from rural coastal Kenya reported home delivery estimates of about 26% in the general population [32]. This is lower compared to estimates from other sSA settings which range from 33% to 94% [9,10,14–20] but higher than 9% to 25% reported in yet other sSA settings[8,11–13].What is less clear is whether these findings can be generalised to HIV-infected women from the same setting. In this paper, we describe the prevalence and correlates of home delivery amongst HIV-infected women aged 18–49 years attending care at a public health facility in this rural setting in Coastal Kenya.

## Methods

### Study site

This study was conducted at the Comprehensive Care and Research Clinic (CCRC) within Kilifi County Hospital (KCH), a rural public health facility in Coastal Kenya. KCH has a catchment population of more than 280,000 people, majority of whom seek HIV services from the CCRC[33]. HIV care is provided according to the Ministry of Health guidelines, which are largely adopted from the WHO guidelines [34]. Following a Kenyan government declaration, all public health facilities, including KCH, have been providing free maternity services from early 2013[35].

### Study design

A mixed methods design was used, involving both quantitative and qualitative approaches. Quantitative data were collected from an analytical cross-sectional approach. In brief, women attending care at the CCRC between March and June 2015 were recruited and interviewed using a validated questionnaire (n = 425). Eligibility included consenting HIV-infected women aged 18–49 years with a history of a recent birth (within five years prior to the study). A five-year period was selected to reduce recall bias and to provide a more current estimate of home delivery in this setting.

Qualitative data were collected using focused group discussions (FGDs, n = 5). FGDs were carried out at the end of quantitative data collection (after July 2015). In brief, women willing to return to the clinic for the FGDs were purposively sampled from those who participated in the quantitative study.The incremental value of incorporating the qualitative component was to help in interpretation of the quantitative findings from the participants’ experiences and cultural context[36].

### Sampling strategy

A consecutive sampling strategy was used. HIV-infected women coming for HIV care at the CCRC were recruited. Assuming an *a-priori* 63% prevalence estimate of home delivery [37], and adjusting for 20% non-response, we estimated that 448 participants will provide prevalence estimate with a precision of +/-5% at 95% confidence levels[38]. During collection of quantitative data women willing to return to the clinic for FGDs were further approached to participate in FGDs. A list of sixty-one women satisfying the criteria was generated. Of these, thirty participants matched by place of delivery (at home [n = 12] and at health facility [n = 18]) were identified, invited and participated in the FGDs.

### Sources of data

An interviewer-administered questionnaire was used to collect quantitative data including socio-demographic, clinical and obstetric history indicators. Socio-demographic indicators included date of birth, marital status, education level, religion and distance to health facility (estimated using Arc Info [Arc Catalog version 9.2, Esri, UK]). Clinical and obstetric history indicators included parity, year of birth of lastborn child (hereafter referred as the reference child), ANC attendance, husband company during ANC visit, gestation at first ANC visit, previous home delivery, whether mother received prevention of mother to child transmission of HIV infection(PMTCT) interventions and duration of highly active antiretroviral therapy (HAART) for the mother. All socio-demographic, clinical and obstetric indicators except age were collected with reference to the time the mothers had their latest birth (reference child). During data collection, questionnaires underwent continuous quality checks and validation for completeness and consistency. Data capture was done in Epi Info 7 (7.1.4.0 [CDC, Atlanta, USA]).

Qualitative data were collected using FGDs (n = 5). Each FGD included between four to eight participants with the overall total number of FGD participants being thirty. FGDs were conducted in the local (Swahili) language using open-ended guides (S2 File). Discussions generally lasted about one hour and were audio-recorded after obtaining informed consent.

### Data analysis

Quantitative data analyses were done using R statistical software (version 3.0.2, Vienna, Austria) [39]. Distribution of baseline characteristics, including socio-demographic, clinical and obstetric indicators, were assessed and presented as frequencies and percentages. The prevalence of home delivery was estimated as the proportion of women who reported to have delivered at a place other than a clinical set up and in the absence of a skilled birth attendant. Binomial 95% confidence intervals (CI) were provided. In addition, multivariable logistic regression analysis using a stepwise model building approach was done. Crude and adjusted odds ratios (aOR), 95% CI and p-values were presented.

For the qualitative analyses, audio files from FGDs were transcribed in the original language (Swahili), translated to English, back-translated into Swahili and independently verified for consistency. The English transcripts were coded in QSR NVivo 11using a thematic analysis approach[40]. An analytic framework was adopted where hospital delivery was directly compared to home delivery and any factor that encouraged the choice of home delivery was immediately considered a barrier to hospital delivery and vice versa. This approach was taken to account for the fact that probes were provided directly about home delivery with an aim of having a better understanding why the participants chose one over the other[36,40,41]. Initial coding was guided by major themes from the interview guide. However new codes and themes were developed on the basis of the data. Excerpts and analytical memos were reviewed to identify common themes and variant views. During a second round of analysis, codes representing similar themes were collapsed to develop fine codes. Illustrative verbatims that represent each theme were chosen and presented [36,40,41].

### Ethical considerations

This study received ethical approval from the Pwani University Ethics Review Committee (Reference No. ERC/MSc/026/2014). Further, institutional authorization was granted by the Kilifi County Research Committee (REF: DOH/KLF/RESCH/VOL. 1/17). Written informed consent was obtained from all the study participants.

## Results

### Quantitative results

#### Characteristics of participants

**Fig 1:**
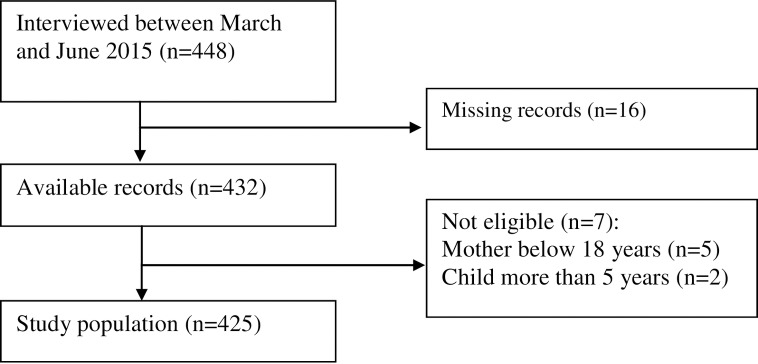
Flow chart showing recruitment of HIV infected women who had a recent delivery and attending care at a rural HIV clinic in Coastal Kenya.

Of the 425 participants included in the analyses (Fig 1), 285 (67.1%) were on HAART at birth of the reference child. Majority of the participants were married in monogamous relationships (278[65.4%]), had attained at least primary education (327[76.9%]), practiced the Christian faith (290[68.2%]), were unemployed (218[51.3%]), lived ≤10 kilometers from the hospital(272[64.0%]), had a parity of ≤4(353[83.0%]), made ≥3 ANC visits during pregnancy for reference child (289[68.0%]), were not accompanied by their spouses for any ANC visit when they were pregnant with reference child (339[79.7%]) and reported a history of a previous home delivery (214[50.3%])(Table 1).

**Table 1:** Characteristics of HIV-infected women with a recent (within 5 years) birth and attending care at a rural HIV clinic in Coastal Kenya (N = 425).

Characteristics	Description/ categories	Not on HAART(n = 140)	On HAART(n = 285)
Age (years)	Median[IQR]	29.4 [25.2–35.2]	30.4 [27.2–36.6]
Age group (years)	18–24	30 [21.4]	45 [15.8]
	25–34	74 [52.9]	145 [50.9]
	35–49	36 [25.7]	95 [33.3]
Marital status	Single	10 [7.1]	31 [10.9]
	Married, monogamous	93 [66.4]	185 [64.9]
	Married, polygamous	20 [14.3]	24 [8.4]
	Divorced/Separated/Widowed	17 [12.1]	45 [15.8]
Education	No schooling	39[27.9]	55[19.3]
	Primary	87[62.1]	173[60.7]
	Secondary/College/Higher	13[9.3]	54[19.0]
	Missing	1[0.7]	3[1.1]
Religion	None	19[13.6]	28[9.8]
	Christian	89[63.6]	201[70.5]
	Muslim	32[22.9]	56[19.7]
Occupation	Unemployed	81 [57.9]	137 [48.1]
	Employed/ informal trading/business	59 [42.1]	148 [51.9]
Distance to hospital (km)	Median[IQR]	7.8 [2.2–13.0]	7.4 [2.2–13.3]
Distance to hospital (km)	<5.0	52 [37.1]	120 [42.1]
	5.0–10.0	38 [27.1]	62 [21.8]
	>10.0	45 [32.1]	96 [33.7]
	Missing	5 [3.6]	7 [2.5]
Parity	Median[IQR]	2 [1–4]	3 [2–4]
Parity	1–2	73 [52.1]	123 [43.2]
	3–4	42 [30.0]	115 [40.4]
	> = 5	24 [17.1]	46 [16.1]
	Missing	1 [0.7]	1 [0.4]
ANC visits	Median[IQR]	4 [2–5]	4 [3–5]
ANC visits	None	14 [10.0]	19 [6.7]
	1–2	21 [15.0]	27 [9.5]
	3–4	55 [39.3]	115 [40.4]
	> = 5	37 [26.4]	82 [28.8]
	Missing	13 [9.3]	42 [14.7]
Male company during ANC—ever?	*No	114 [81.4]	228 [80.0]
	Yes	26 [18.6]	53 [18.6]
	Missing	0 [0.0]	4 [1.4]
Year of birth, last born (reference) child	2010–2011	53 [37.9]	65 [22.8]
	2012–2013	61 [43.6]	110 [38.6]
	2014–2015	26 [18.6]	110 [38.6]
History of previous home deliveries	Never delivered at home	24 [17.1]	72 [25.3]
	First delivery	40 [28.6]	63 [22.1]
	History of previous home delivery	74 [52.9]	140 [49.1]
	Missing	2 [1.4]	10 [3.5]

IQR (Interquartile ranges)

*Includes mothers who never attended antenatal care, ANC (n = 18)

### Prevalence and correlates of home delivery

Of the 425 participants included in the analysis, 108 (25.4% [95% CI: 21.3–29.8]) reported to have delivered the reference child at home. In the multivariable model, education status, history of a previous home delivery and being on HAART were independent predictors of home delivery. Mothers with no formal education had increased odds of reporting a home delivery compared to those with a secondary/higher education (adjusted odds ratios, aOR [95% C.I], p-value: (12.4 [3.4–46.0], p<0.001). Equally, mothers reporting a history of a previous home delivery had increased odds of reporting a home delivery, compared to those who had never previously delivered at home (2.7 [1.2–6.0], p = 0.019). Lastly, mothers on HAART had decreased odds of reporting a home delivery compared to those who were not on HAART at the time of delivery of the reference child (0.4 [0.2–0.8], p = 0.006). After adjusting for other covariates, the effects of maternal age, religion, occupation status, distance to hospital, ANC visits and calendar year of birth of reference child were attenuated towards the null (Table 2).

**Table 2:** Risk factors associated with home delivery amongst HIV-infected women with a recent (within 5 years) birth and attending care at a rural HIV clinic in Coastal Kenya.

Characteristic	Category	Home delivery [%]	Crude OR [95% CI]	p-value	Adjusted OR, [95% CI]	p-value
Age (years)	18–24	13 [17.3]	Ref		Ref	
	25–34	54 [24.7]	1.6 [0.8–3.1]	0.194	1.4 [0.6–3.3]	0.454
	35–49	41 [31.3]	2.2 [1.1–4.4]	0.030	1.3 [0.5–3.4]	0.579
Marital status	Single	7 [17.1]	Ref			
	Married, monogamous	75 [26.9]	1.8 [0.8–4.2]	0.180		
	Married, polygamous	7 [15.9]	0.9 [0.3–2.9]	0.885		
	Divorced/Separated/Widowed	19 [30.7]	2.1 [0.8–5.7]	0.125		
Education	No schooling	48 [51.1]	16.4 [5.5 –48.8]	<0.001	12.4 [3.4–46.0]	<0.001
	Primary	55 [21.2]	4.2 [1.4 –12.1]	0.007	4.0 [1.1–13.9]	0.030
	Secondary/College/Higher	4 [5.9]	Ref		Ref	
Religion	None	18 [38.3]	Ref		Ref	
	Christian	72 [24.8]	0.5 [0.2 –1.0]	0.055	0.8 [0.4–1.8]	0.602
	Muslim	18 [20.4]	0.4 [0.1 –0.9]	0.028	0.6 [0.2–1.6]	0.337
Occupation status	Unemployed	69 [31.7]	Ref		Ref	
	Employed/Trader	39 [18.8]	0.5 [0.3–0.8]	0.003	0.8[0.5–1.4]	0.494
Distance to hospital (km)	<5.0	32 [18.6]	Ref		Ref	
	5.0–10.0	30 [30.0]	1.9 [1.1–3.3]	0.032	1.1 [0.5–2.1]	0.846
	>10.0	40 [28.3]	1.7 [1.0 –2.9]	0.042	1.3 [0.7–2.5]	0.453
Parity	1–2	41 [20.9]	Ref			
	3–4	44 [28.0]	1.5 [0.9 –2.4]	0.122		
	> = 5	22 [31.4]	1.7 [0.9 –3.2]	0.078		
ANC visits	None	14 [42.4]	3.2 [1.4–7.5]	0.005	1.5 [0.6–4.2]	0.396
	1–2	17 [35.4]	2.4 [1.1 –5.1]	0.021	1.8 [0.7–4.5]	0.196
	3–4	49 [28.8]	1.7 [1.0 –3.1]	0.046	1.2 [0.6–2.4]	0.534
	> = 5	22 [18.5]	Ref		Ref	
Spouse male company during ANC—ever	No	91 [26.6]	Ref			
	Yes	17 [21.5]	0.8 [0.4 –1.4]	0.352		
Year of birth for the last born	2010–2011	35 [29.6]	Ref		Ref	
	2012–2013	50 [29.2]	1.0 [0.6–1.6]	0.938	0.9 [0.4–1.7]	0.653
	2014–2015	23 [16.9]	0.5 [0.3 –0.9]	0.017	0.7 [0.3–1.4]	0.297
Previous home delivery	Never delivered at home	13 [13.5]	Ref		Ref	
	First delivery	18 [17.5]	1.4 [0.6–2.9]	0.446	1.1 [0.4–2.9]	0.905
	History of previous home delivery	74 [34.6]	3.4 [1.8 –6.5]	<0.001	2.7 [1.2–6.0]	0.019
On HAART	No	58 [41.4]	Ref		Ref	
	Yes	50 [17.5]	0.3 [0.2–0.5]	<0.001	0.4 [0.2–0.8]	0.006

ANC (Antenatal Care); HAART (Highly Active Antiretroviral Therapy); OR (Odds Ratio) and CI (Confidence Interval)

Further exploration amongst those who reported to have delivered at home suggests that majority reported sudden onset of labour (78[36.1%]) and further distance from the hospital (47[21.8%]) as the main reasons for home deliveries (Table 3).

**Table 3:** Reasons for home delivery among HIV infected women who delivered their reference child at home (n = 108).

Reason	N = 108	%**
Sudden labour pains	78	72.2
Far distance to hospital	47	43.5
No previous complications	19	17.6
Being at home alone	18	16.7
High cost	13	12.0
Preference for TBA	12	11.1
Facility not open	7	6.5
Disallowed by husband/family	7	6.5
Don’t trust health workers	5	4.6
Unnecessary	3	2.8
No female health care provider	2	1.9
*Other reasons	5	4.6

^*^Heavy rains, delivered en-route to facility, late night

^**^Overall, 108 women delivered at home, but some gave multiple reasons

### Qualitative results

Based on a thematic analysis, four major themes were identified; a) participants’ preference for hospital delivery over home delivery, b) barriers to facility delivery services c) facilitators of health facility delivery and d) non-facilitators of health facility delivery (Table 4).

**Table 4:** Summary of qualitative findings illustrating derivation of the themes, sub-themes and exemplifying participant verbatims.

Theme	Subtheme	Exemplifying statement
Preference for hospital delivery over home delivery	Skilled birth attendants in hospital	I made a decision of delivering in hospital because we have doctors (Service providers) with delivering skills
	Birth attendants at home unskilled in PMCT	When the child is born and there are no skills for separating the umbilical cord “I am finished”. The viruses pass from the mother to the child.
	Labor mismanagement and harassment	…she (Birth attendant at home) beats you and forces you to push and some time she inserts her hand
	Stigma and discrimination at home	…you have delivered at home, when a person knows your status it brings stigmatization, people start gossiping/talking and it affects you
	Education and advice from health workers	. . .you are advised you get to know that how you will stay with the child like were told to breast feed the baby for six months then you don't breastfeed again so if you are at home you won't know
	No equipment at home to conduct delivery	I delivered at home but I did not see any equipment it was the same situation of using bare hands…
	Good reception at hospital	I was received well and given a bed
Barriers to facility delivery services		
*i) Consumer associated barriers*	Lack of finances	I don't have money how will I go there (hospital)
	Rapid progression of labour	When I was going to sleep I started feeling like am in pain…like some sort of illness …after like thirty minutes I delivered…..
	Previous safe home delivery	I delivered without any problem at home
	Being alone at home	There is no one to take me to hospital. . .may be my husband has gone to work and I am in labour. . .I delivered …at home alone.
	Not attending ANC	Failure to attend ANC during pregnancy, also not having the ANC mother child booklet contribute to home delivery….
	Inadequate knowledge	. . .because the date of delivery (given during ANC) is inaccurate so it is unreliable. . .
	Fear of being tested for HIV	Some of us fear being tested because we don't trust ourselves
	Travelling	One could be travelling in a vehicle and labour pain starts. . .you will deliver in the vehicle. . .
*ii) Health care provider associated barriers*	Delays in providing services	I told them I was in pain they told me…mother you are in a hurry this child is not due…
	Stigma and discrimination	There is discrimination in maternity, when they look at your book and see you are living with HIV, they leave you, when a HIV negative woman comes they attend to her. . .
	Negative attitude of service providers	. . . I said if I get pregnant again I will not go back to hospital(to deliver). . . . .how could I give birth on the floor yet the health care provider is there and I had told her the baby was due and she sent me away like a dog. . . .
	Unavailability of service providers	In hospital even doctors are not there so we are going to hospital to do what…
	Breach of confidentiality	I go and give birth in hospital… and see them again overcrowded in the room I better deliver at home
	Being attended by a male provider	…its only men who will assist you…you feel shy to go and be naked before many men…
*iii) Structural barriers*	Long distance to hospital	I like so much to go and give birth at the hospital but now the place is far
	Lack of means of transport	They try to look for means of transport to take you to the hospital and they don't succeed so you give birth at home
	Poor road network	…the road condition is bad…
Facilitators of health facility delivery	Good relationship with service providers	. . .because my cousin was there (hospital) who knows my HIV status and he attended to me very well…
	Services being free	…they serve us well when we can't afford… that is when a mother comes to give birth and she can't afford
Non-facilitators of health facility delivery	Birth attendants at home provide comfort during delivery	…if you have delivered there at home you are comforted…
	Availability of trained TBAs at home	…they were trained even to do abdominal massage and receiving the baby. . .they can do it (assisting in delivery) like there (hospital)
	Being uncooperative to service provider	…he (service provider) tell you do this and you do not do he becomes harsh you see may be he gets bored until he leaves you there

**a) Participants’ preference for hospital delivery over home delivery**. In general, most of the participating mothers preferred hospital delivery over home delivery, and provided several reasons for this. For instance, they reported that in the hospital there were skilled attendants capable of dealing with complications if they arose during child birth unlike at home. Some mothers felt it was good to deliver in a health facility in order to prevent transmission of HIV infection from mother to child because at home the attendants were not skilled in PMTCT. Home delivery was perceived by the participants to be disadvantageous compared to facility delivery as it was viewed to be associated with labor mismanagement and harassment since the people who assist during child birth at home are not skilled enough in labor management. Participants felt they were being stigmatized and discriminated due to their HIV status when they deliver at home hence opting for health facility delivery. A good number of participants preferred health facility delivery because at the hospital they would get advice and information on various aspects of maternal and child health from health care providers, such education and advice is not available at home. In addition there were no important equipments at home for conducting delivery unlike in hospital. Participants also preferred health facility delivery because at the hospital one is received well on arrival to deliver (Table 4).

**b) Barriers to facility delivery services**. Despite what seemed to be a unanimous endorsement of hospital delivery (due to better quality of care and PMTCT), a significant proportion of participants gave birth at home. There was an interest therefore to understand the factors that contributed to this decision. The thematic analysis indicated that these factors could be grouped into three major themes; i) consumer associated barriers, e.g. lack of finances ii) health care provider associated barriers, e.g. delays in providing services and iii) structural barriers, e.g. long distance to hospital (Table 4).

**c) Facilitators of health facility delivery**. Participants felt certain factors encouraged them to have a bias towards hospital delivery. Participants thought it was easy to deliver in the hospital if one has a good relationship with the care providers since they would serve her well. For a few of the participants, it was even better if the health care provider was a relative. Provision of delivery services free of charge was seen as an incentive for mothers to deliver in hospital.

**d) Non-facilitators of health facility delivery**. Participants reported certain disincentives for facility delivery. They felt the attendants at home during delivery provide comfort and stay with the mother throughout hence an incentive for one to deliver at home. In addition presence of trained TBAs at home was a disincentive for health facility delivery since such attendants were capable of providing delivery care just like in the hospital. For some participants, failing to cooperate with service providers at the hospital would not facilitate health facility delivery as the provider may end up not attending to the mother (Table 4).

## Discussion

Despite a strong endorsement against home delivery and high coverage of ANC visits during pregnancy, a quarter of HIV-infected women attending care in a rural public health facility in Coastal Kenya reported to have delivered at home. Mothers without formal education and those with history of home delivery were more likely to deliver at home. On the other hand, mothers on HAART were less likely to deliver at home.

Home delivery in this setting was lower compared to Western Kenya and Nairobi/Nyanza regions, which reported estimates of 53% and 31% respectively in the HIV infected populations between 2008 and 2011[25,26]. Our estimate was also lower compared to what has been previously reported in other sSA settings[21,23]. The Western Kenya studies were carried out between 2008 and 2011, and before the implementation of free maternity services in 2013[35], which may explain the high estimates in home delivery from Western Kenya. Indeed, our data confirm an overall decline in home deliveries by calendar years (calendar years: home deliveries [2010–2013:85%; 2014–2015:23%]), though this did not come out as an independent correlate of home deliveries in the multivariate analyses. The qualitative findings equally suggest lack of finances as a barrier to facility delivery services while provision of free delivery services facilitates facility delivery.

Our estimates are however comparable to the 26% prevalence recently reported from the general population in this setting[32]. Efforts aiming to reduce mother to child transmission(MTCT) of HIV infection have traditionally tended to emphasize health-facility delivery[42]. It is therefore interesting that home deliveries amongst HIV-infected women in this setting were comparable to those from the same general population, and not lower as expected [32].Towards the expiry of the millennium development goals in 2015, the Kenyan government stepped up efforts to scale up hospital deliveries by introducing free maternity services in public hospitals in 2013[35].This intervention targeted all mothers regardless of HIV status, and may thus explain the observed comparability in home delivery estimates. A recent study from Zimbabwe has reported similar findings; that the likelihood of facility-based delivery is not associated with maternal HIV status[22]. In addition literature suggests that risk factors for home delivery among women in general population are comparable to those in HIV-infected women [11,27,30,31].

Women with a formal education were less likely to deliver at home compared to those who had no formal education. These findings were complemented with the qualitative results, which also revealed inadequate knowledge on pregnancy and child birth as a barrier to facility based delivery. It is possible that educated women are more informed and therefore could readily appreciate the need to attend health facility delivery compared to those without a formal education. Studies in other settings have reported similar findings[4–6,17,26,31,43,44].

Mothers with a history of a home delivery were more likely to continue delivering at home. Equally, in the qualitative approach a previous safe home delivery came out as a barrier to facility delivery. This is possibly because many women in rural sSA setting view health facility delivery as being appropriate only for those with birth-related complications [4,45]. A study from rural Ghana reported similar findings that women with a history of a home delivery were more likely to continue delivering at home[8]. Indeed, and following uneventful home deliveries, women culturally probably take this as a confirmation that they are capable of successfully going through child birth at home, hence unlikely to seek skilled care in subsequent pregnancies[8].

Our analyses show that being on HAART was a deterrent factor for home delivery, emphasizing the importance of ensuring all HIV infected pregnant women are started on HAART at diagnosis of HIV infection[34]. It is possible that being on HAART brings the women closer to the health care services through regular clinic appointments for drug refill and other linked comprehensive services including adherence counseling, birth planning and preparedness in the context of their HIV positive status.

Interestingly, distance to health facility did not predict place of delivery after controlling for other factors, though it came out as a barrier to hospital delivery among other structural barriers in the qualitative results. Most previous studies on choice of place of delivery found longer distance to be a significant predictor for home deliveries [12,44]. In recent years there has been an increase in community level health facilities in Kenya, funded by devolved governance funds. Many women have a health facility within reach and this could explain why distance to health facility was not a significant predictor of place of delivery in this setting. In addition, poor state of roads came out as a barrier to hospital delivery in the qualitative findings suggesting that the challenge is more of an infrastructural access to health facilities rather than physical distance in kilometers.

An important strength of this study is the use of a mixed methods approach which provided context and deeper understanding of the quantitative findings. However, our findings are to be interpreted in light of several limitations. Firstly, this was a hospital-based study. There could have been more women in the community who delivered at home, suggesting that we may have underestimated the prevalence of home deliveries in this population. Secondly, our findings may not apply, and cannot be generalised to more developed countries. This is because home deliveries are supervised by skilled attendants, and even encouraged, in some developed settings.

## Conclusion

A quarter of HIV-infected women delivered at home, which is comparable to estimates reported from the general population in this rural setting, and much lower than estimates from other sSA settings. A tailored package of care targeting women with no formal education and with a history of a previous home delivery, coupled with interventions towards scaling up HAART and improving the quality of maternal care in HIV-infected women may positively contribute to a decline in home delivery and subsequent maternal mortality in this setting.

## Ethical approval

All procedures performed in this study were in accordance with the ethical standards of the institutional and/or national research committee and with the 1964 Helsinki declaration and its later amendments or comparable ethical standards. The study received ethical approval from the Pwani University Ethics Review Committee (Reference No. ERC/MSc/026/2014). Further, institutional authorization was granted by the Kilifi County Research Committee (REF: DOH/KLF/RESCH/VOL. 1/17).

## Supporting information

S1 File Interview guide English translation. (DOCX)

S2 File Interview guide Swahili. (DOCX)
