# Supplementary material for: Prevalence and correlates of home delivery amongst HIV-infected women attending care at a rural public health facility in Coastal Kenya
Source: PLoS One. 2018 Mar 20;13(3):e0194028. doi: 10.1371/journal.pone.0194028 (PMC5860701; doi:10.1371/journal.pone.0194028)
Supplement: S1 File — (DOCX) [file pone.0194028.s001.docx]

Interview guide_ English Translation

| *Theme.* | *Questions.* |
| --- | --- |
| Perceived quality of delivery care in health facilities | 1. What are your views on facility delivery services 2. Are you satisfied with delivery services? |
| What are the women’s’ views on home delivery? | 1. What are the effects of home delivery 2. What are reasons for home delivery 3. What challenges does one face when delivering at home |
| What are women’s’ views on facility delivery? | 1. What are the reasons for choosing a facility delivery 2. What are the obstacles to accessing facility delivery services |
| Are they aware about MTCT of HIV? | 1. How can MTCT of HIV be prevented |
