# Supplementary material for: Prevalence and correlates of home delivery amongst HIV-infected women attending care at a rural public health facility in Coastal Kenya
Source: PLoS One. 2018 Mar 20;13(3):e0194028. doi: 10.1371/journal.pone.0194028 (PMC5860701; doi:10.1371/journal.pone.0194028)
Supplement: S2 File — (DOCX) [file pone.0194028.s002.docx]

Interview guide_ Swahili

| *Theme.* | *Questions.* |
| --- | --- |
| Dhana kuhusu ubora wa huduma za uzazi katika vituo vya afya | 1. Mna maoni gani kuhusu huduma za uzazi katika vituo vya afya 2. Mna ridhika na hizi huduma za uzazi? |
| Wanawake wana maoni gani kuhusu kuzaa nyumbani? | 1. Kuzaa nyumbani kuna athari gani 2. Sababu za kuzaa nyumbani ni zipi 3. Ni changamoto gani mtu hukumbana nazo anapozaa nyumbani |
| Wanawake wana maoni gani kuhusu kuzaa katika kituo cha afya? | 1. Ni sababu zipi za kuchagua kujifungua katika kituo cha afya 2. Ni pingamizi gani zinamzuia mtu kupata huduma za uzazi za hospitali |
| Je wana ufahamu kuhusu uambukizaji wa virusi vya ukimwi kutoka kwa mama hadi kwa mtoto? | 1. Uambukizaji wa virusivya ukimwi kutoka kwa mama hadi kwa mtoto kunaweza kuzuiliwa namna gani |
